# Basilicata-Akhtar Syndrome: Unraveling an Ultrarare Cause of Developmental Delay

**DOI:** 10.7759/cureus.67041

**Published:** 2024-08-16

**Authors:** Vishwanath Kulkarni, Shiji Chalipat, Aryan Gupta, Akanksha Bhosle, Mridu Bahal

**Affiliations:** 1 Pediatrics, Dr. D. Y. Patil Medical College, Hospital and Research Centre, Dr. D. Y. Patil Vidyapeeth (Deemed to be University), Pune, IND

**Keywords:** whole-exome sequencing, hypotonia, dysmorphism, global development delay, human genetics and epigenetics

## Abstract

There is still more to learn about the etiology of extremely uncommon developmental disorders. A heterozygous or hemizygous pathogenic variation in male-specific lethal 3 (MSL3) causes the uncommon X-linked condition known as Basilicata-Akhtar syndrome, which is characterized by a global developmental delay that is evident from infancy, feeding difficulties, and muscle hypotonia. Thus far, over 40 cases have been documented. Here, we report the first case of Basilicata-Akhtar syndrome in India. A 3-year-old boy presented with global development delay. Physical examination revealed dysmorphism and hypotonia. After whole exome sequencing, exon 8 of the MSL3 gene on chromosome X showed evidence of a hemizygous single base pair deletion.

## Introduction

The X chromosome harbors male-specific lethal 3 (MSL3), which encodes for a subunit of the chromatin-associated MSL complex [1,2]. The MSL complex is an essential epigenetic regulator in humans and flies, mediating global histone H4 lysine-16 acetylation (H4K16ac) [1-4]. The core MSL complex in mammals comprises MSL1, MSL2, MSL3, and males absent on the first [2-5]. Recently, MSL3 was discovered as the fundamental genetic etiology of the Basilicata-Akhtar syndrome (MIM 301032), a newly discovered X-linked neurodevelopmental disease that is equally prevalent in men and women and was previously found to be a potential gene in the deciphering developmental disorder study [6,7]. Neurodevelopmental diseases have been linked to mutations in epigenetic regulators, which may modify the chromatin landscape [8]. Thus far, more than 40 Mendelian disorders that result from mutations in the epigenetic machinery and are linked to neurological dysfunction have been found [9-11]. The final form and functionality of the adult brain and the entire organism are influenced by fluctuations in the histone acetylation equilibrium, which are caused by the interaction between acetylase and deacetylase functions during development [12-15]. Global developmental delay, delayed speech, feeding difficulties in infancy, facial dysmorphism, muscle hypotonia, and spasticity are common clinical symptoms in affected individuals [7].

## Case presentation

A 3-year-old male boy was brought with complaints of delayed milestone attainment. On detailed history taking, the language domain (development quotient (DQ) of 20%) was found to be more affected than the motor domain (DQ of 40%). Ventriculomegaly was detected during the prenatal scan. Both natal and postnatal history were insignificant. He was born to parents who were not closely related, and the family history was irrelevant.

**Figure 1:**
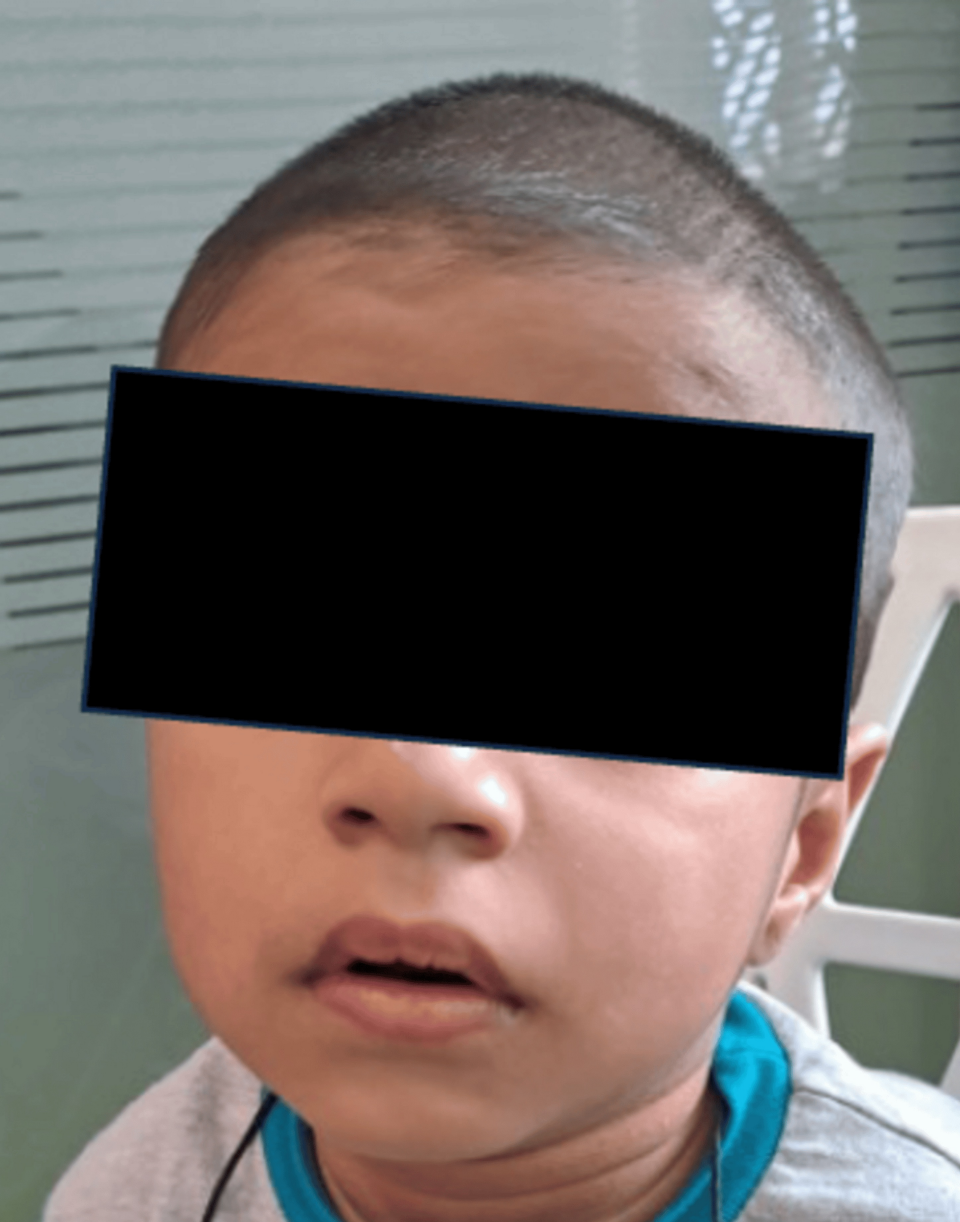
Facial dysmorphism

**Figure 2:**
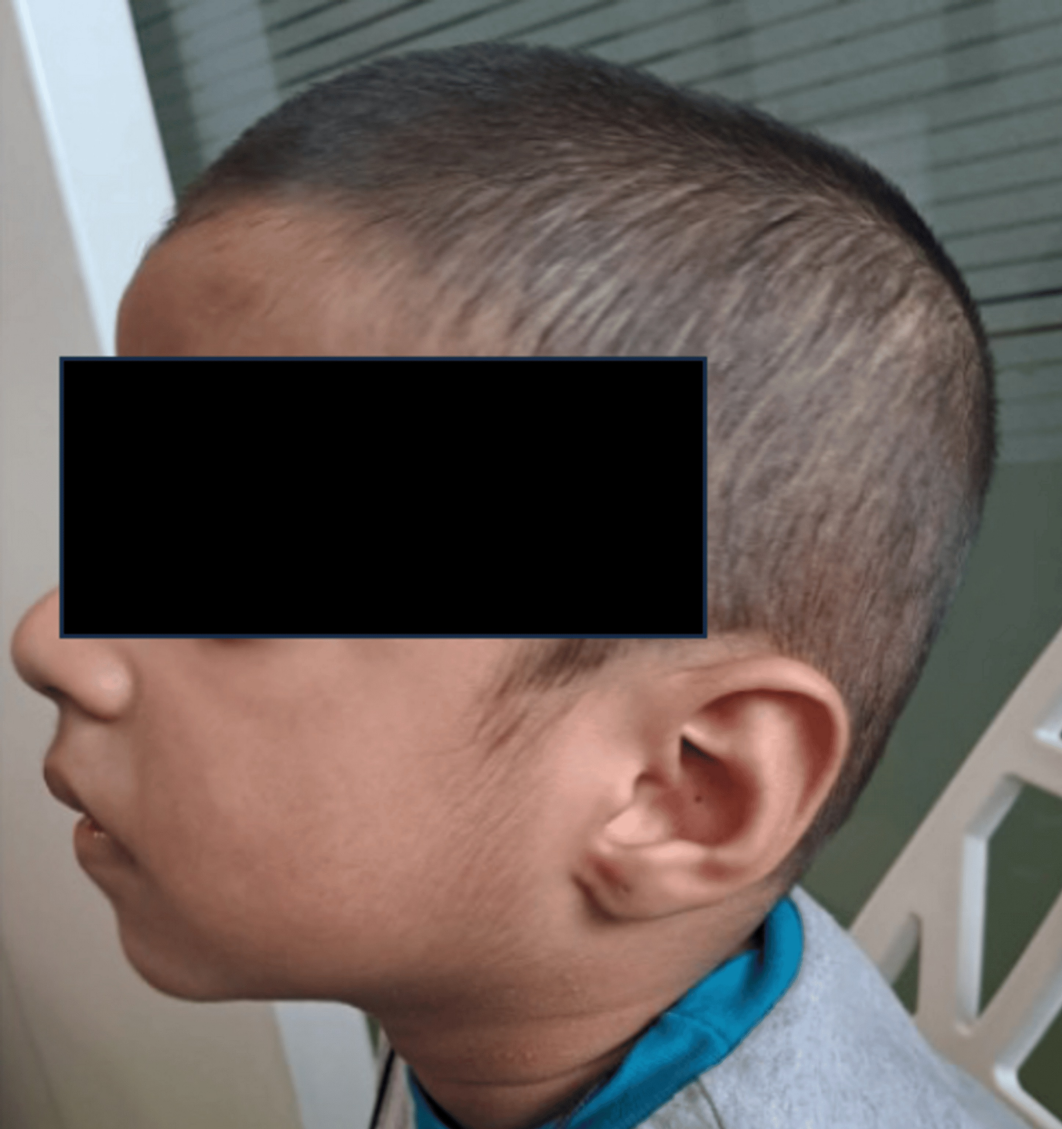
Low-set and posteriorly rotated ears

On examination, facial dysmorphism was present. He had downslanting palpebral fissures, epicanthic folds, a broad nasal bridge, posteriorly rotated low-set ears, and a downturned angle of the mouth (Figures 1, 2). No neurocutaneous markers were found. He was conscious and alert during the central nervous system examination, and the cranial nerve examination was within normal limits. Motor system examination revealed hypotonia in all four limbs, brisk deep tendon reflexes, and an extensor planter response. The rest of the examinations were within normal limits.

**Figure 3:**
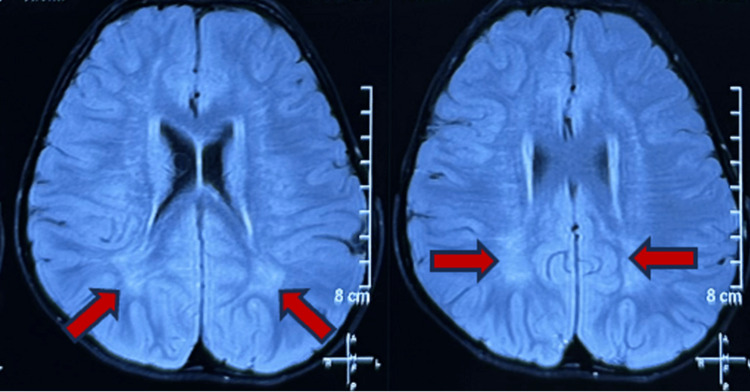
Peritrigonal and subcortical white matter hyperintensities (red arrows)

A clinical diagnosis of syndromic developmental delay was made based on history and examination. Thyroid function and complete blood count were performed, and the results were normal. An MRI of the brain was done, which revealed T2 and fluid-attenuated inversion recovery peritrigonal and subcortical white matter hyperintensities (Figure 3).

The results of the brainstem-evoked response audiometry were within normal bounds. As part of the syndromic work-up, abdominal ultrasonography and echocardiography were performed, and the results were normal. Whole exome sequencing was planned, and it initially showed no pathogenic variants. The sample was sent for reanalysis, which revealed a hemizygous single base pair deletion in exon 8 of the MSL3 gene on chromosome X (p.Ser297ValfsTer19; ENST00000312196.10), which was a likely pathogenic variant known to cause Basilicata-Akhtar syndrome.

The patient was started on physiotherapy and speech therapy. Parents were given genetic counseling and counseled about Sanger sequencing, but since they were not planning to have another child, they chose not to proceed. Advice on the prognosis and likelihood of recurrence in subsequent pregnancies was given to the parents.

## Discussion

Basilicata-Akhtar syndrome is an ultrarare cause of syndromic development delay, resulting from the loss of the MSL3 gene, which results in pronounced loss of H4K16ac. This indicates that complicated developmental processes, such as those in the brain, may be regulated in humans through MSL-mediated H4K16ac. Reconciling it with traditional X-chromosomal inheritance models is difficult because it affects both heterozygous females and hemizygous men with similar symptoms [7].

The biggest cohort of people with an X-linked MSL3-related condition to date was documented by Brunet et al. in relation to 25 people (15 males and 10 females) with a disease that caused hemizygous or heterozygous variations in MSL3. With the exception of one individual whose mother had germline mosaicism despite having a normal phenotype, most instances (n = 21) were either verified or determined to be de novo according to family history (n = 3). This suggests that the majority of mutations are de novo. Developmental delay was present in all patients, with most having global delays. Intellectual disability was also seen. Compared to expressive language, receptive language abilities appeared to be more developed. A couple of patients showed signs of developmental regression (verbal and cognitive in one and motor regression in the other). Some patients had autism spectrum disorder (50%). Other behavioral abnormalities included attention-deficit/hyperactivity disorder, obsessive-compulsive disorder, aggressive behavior, anxiety, and self-injurious behavior [16]. Our patient had a global development delay with predominant involvement of the language domain and more affection for expressive language. Most of the patients described also had a history of abnormal muscle tone, predominantly hypotonia, but some also had spasticity. It was discovered that while spasticity seemed to develop later in life as one aged, hypotonia was primarily noticeable in infancy [16]. Hypotonia was present in our patient. The literature detailed a wide range of movement disorders, none of which were seen in our patient, including dystonia, bradykinesia/hypokinesia, and ataxia. Few patients had a history of seizures, but these were successfully controlled with antiseizure drugs. Our patient did not have any seizures. The majority of patients had facial dysmorphism, which comprised coarse facial features, low-set or posteriorly rotated ears, a round face, a large nasal bridge, epicanthal folds, hypertelorism, and a high forehead. High nasal bridges, overbites, small palates, micrognathia, aberrant dentition, and arching brows were among the other characteristics [16]. Among these, our patient had a broad nasal bridge, epicanthic folds, hypertelorism, and low-set ears rotated posteriorly. The presence of macrocephaly, polyhydramnios, constipation, gastroesophageal reflux, urinary symptoms (hydroceles, hydronephrosis, pyeloureteral junction, and urine retention), vision abnormalities, conductive and/or sensorineural hearing loss, skeletal abnormalities (pes planus, plagiocephaly, and pectus carinatum), and respiratory and cardiovascular complaints were among the other findings observed in the study [16]. Among the aforementioned, constipation and gastroesophageal reflux were seen in our case. The study's MRI abnormalities included nonspecific white matter abnormalities, mild expansion of external cerebrospinal fluid spaces, hypoplasia of the inferior vermis, and variable dilatation at the trigone of the lateral ventricle [16]. In our case, subcortical white matter hyperintensities were seen.

Even though no specific treatment is available, numerous consequences related to the condition can arise. These complications can be addressed symptomatically. The clinical phenotypic definition of this MSL3-associated illness allows for formulating the following recommendations for clinical therapy and follow-up. Affected individuals should be directed to the following after being diagnosed: (1) a pediatric ophthalmologist since visual impairment was common and early treatment might be necessary to prevent amblyopia; (2) a pediatric otolaryngologist since cochlear implants or hearing aids should be used to treat hearing impairment as soon as possible; (3) gastroenterological assessment for gastroesophageal reflux and feeding problems; (4) for the purpose of identifying structural abnormalities that could be a risk factor for urinary tract infections and/or subsequent renal damage, nephrology assessment, which includes kidney ultrasonography, is advised. Prophylactic treatment and subsequent evaluation by a pediatric nephrologist may be necessary; (5) assessment of the skeletal system for deformities that would need orthopedic care; and (6) as movement problems, seizures, behavioral abnormalities, and developmental delays account for the majority of the disease burden, regular neurological and neurodevelopmental follow-up is necessary [16].

## Conclusions

Basilicata-Akhtar syndrome is a rare genetic disorder, and to the best of our knowledge, this is the first case report published in India. It is characterized by intellectual disabilities, developmental delays, distinctive facial features, and other symptoms. Future studies should aim to characterize the clinical spectrum of MSL3 mutations further and investigate potential genotype-phenotype correlations. Ongoing research is necessary to understand the underlying genetic pathways and find appropriate treatments and interventions for individuals with Basilicata-Akhtar syndrome.
